# A Case of 17β-Hydroxysteroid Dehydrogenase Type 10 (HSD10) Disease Caused by a Novel Variant Presenting With Rapidly Progressive Cardiomyopathy Triggered by Viral Infection

**DOI:** 10.7759/cureus.93552

**Published:** 2025-09-30

**Authors:** Ryusei Kubo, Yoichi Iwamoto, Sayaka Ajihara, Kei Murayama, Akira Ohtake, Sumie Fujinuma, Koki Sugiyama, Hirotaka Ishido, Seigo Korematsu, Satoshi Masutani

**Affiliations:** 1 Pediatrics, Saitama Medical Center, Saitama Medical University, Kawagoe, JPN; 2 Pediatrics and Clinical Genomics, Saitama Medical University Hospital, Iruma, JPN; 3 Diagnostics and Therapeutics of Intractable Diseases, Intractable Disease Research Center, Graduate School of Medicine, Juntendo University, Tokyo, JPN

**Keywords:** cardiomyopathy, hsd10 disease, hsd17b10, mitochondrial disorder, val12met, variant

## Abstract

Human 17β-hydroxysteroid dehydrogenase type 10 (HSD10) is a rare X-linked mitochondrial disorder caused by mutations in the *HSD17B10* gene. It is typically associated with neurodegeneration and cardiomyopathy in severe cases. The neonatal form often has a poor prognosis; however, its clinical spectrum remains unclear. Herein, we present a neonatal-onset case of HSD10 disease with a previously unreported *HSD17B10* variant presenting with early-onset cardiomyopathy triggered by a viral infection. A male infant presented with lactic acidosis and hypoglycemia on the first day of life. Initial treatment improved metabolic status. He was diagnosed with HSD10 disease using a targeted panel of nuclear and mitochondrial genes, which identified a novel hemizygous variant, NM_004493.3: c.34G>A p.(Val12Met). Cardiac function was normal at five months, but neurodevelopmental regression occurred at six months following vaccination. At seven months, viral myocarditis was diagnosed following rhinovirus/enterovirus infection. Echocardiography revealed a reduced left ventricular ejection fraction and ventricular dilation. Despite supportive therapy, cardiac function deteriorated, leading to death at nine months. A patient with a novel *HSD17B10* variant showed early-onset cardiomyopathy in a neonatal case of HSD10 disease and rapid and fatal deterioration of cardiac function. This report highlights the importance of clarifying genotype-phenotype correlations to guide the diagnosis and management of rare mitochondrial diseases.

## Introduction

Human 17β-hydroxysteroid dehydrogenase type 10 (HSD10) disease is a rare X-linked disorder caused by mutations of the *HSD17B10* gene [1]. The 17β-HSD10 protein is encoded by the *HSD17B10* gene, which is mapped to chromosome Xp11.2 [2]. The 17β-HSD10 protein is a multifunctional enzyme involved in branched-chain isoleucine and fatty acid metabolism [3]. It is an essential component of mitochondrial ribonuclease P, which is required for mitochondrial transfer ribonucleic acid (tRNA) processing. This enzyme is expressed in various tissues, especially the liver, heart, and brain [4]. It also has an affinity for amyloid beta protein and is involved in the pathogenesis of Alzheimer's [5].

The clinical symptoms of HSD10 disease include features of mitochondrial dysfunction and, in many cases, progressive cardiomyopathy. The latter is a distinct feature of the HSD10 disease, which is different from typical mitochondrial dysfunction with neurological symptoms, including progressive cognitive and motor decline, epilepsy, and blindness. The Zschocke classification is often used to categorize clinical forms, such as neonatal, infantile, and juvenile forms [1]. The neonatal form has a poor prognosis with severe metabolic acidosis and elevated lactate levels during the neonatal period, followed by progressive cardiomyopathy and death during infancy. The infantile form is the most common course of the disease, presenting with neurological symptoms between six months and two years of age, with death often occurring between two and four years of age. The juvenile form has only been reported in one case. Atypical cases show decreased weight, height, and head circumference, but with normal intellectual and neurological development. The long-term prognosis for this disease form is unknown [6]. Because of the X-linked mode of inheritance, heterozygous females may present with symptoms, such as mild intellectual disability.

In this report, we describe the case of a Japanese male infant with neonatal-onset disease who showed progressive neurodegeneration after vaccination at six months, developed myocarditis and cardiomyopathy at seven months due to a viral infection, and died at nine months. To date, only six cases of neonatal-onset HSD10 disease have been reported [7]. All these cases, including the one described in this report, resulted in death within a few months of birth, indicating a uniformly poor prognosis. A novel variant, NM_004493.3: c.34G>A p.(Val12Met), was identified in the *HSD17B10* gene by sequencing.

This article was previously presented as a meeting abstract at the 60th Annual Meeting of the Japanese Society of Pediatric Cardiology and Cardiac Surgery on July 11, 2024.

## Case presentation

A male infant was born in another hospital at 41 weeks and 0 days of gestation, with a body weight of 3,004 g (-0.6 SD), height of 49.5 cm (-0.2 SD), head circumference of 33.8 cm (0.1 SD), and chest circumference of 31.0 cm. His APGAR scores were 9 and 10 at one minute and five minutes, respectively. On the first day of life, he presented with metabolic acidosis, hyperpnea, and elevated lactate levels and was admitted to the neonatal intensive care unit at our hospital. Laboratory testing on admission showed metabolic acidosis, mild hyperammonemia, and severe hypoglycemia and hyperlactatemia. Laboratory parameters are summarized in Table 1.

**Table 1:** Initial laboratory testing on the first day of life Blood examination revealed metabolic acidosis, mild hyperammonemia, and severe hypoglycemia and hyperlactatemia. Reference ranges are provided for the clinical context.

Parameter	Value	Reference Range	Unit
pH	7.061	7.35–7.45	-
pCO_2_	29.8	35–45	mmHg
HCO_3_	8.4	21–27	mmol/L
Lactate	186	5–20	mg/dL
Ammonia	145	12–66	μg/dL
Glucose	12	70–109	mg/dL
White blood cell	39,200	4,800–18,500	/mL
Hemoglobin	17.4	8.7–13.5	g/dL
Blood urea nitrogen (BUN)	8	8–20	mg/dL
Creatine kinase (CK)	1,375	40–300	IU/L
CK-MB	70.1	0.0–5.0	ng/mL
Aspartate aminotransferase (AST)	142	20–62	IU/L
Lactate dehydrogenase (LD)	353	198–404	IU/L

Additional laboratory findings included elevated levels of tiglylcarnitine or isovalerylcarnitine (C5:1) and glutarylcarnitine (C5-DC) during newborn screening in Japan. While urinary organic acid analysis in HSD10 disease generally shows elevated 2-methyl-3-hydroxybutyrate (2M3HB) and tiglylglycine [1], this case presented with elevated 2,3-dihydroxy-2-methylbutyrate (2,3DH2MB), 3-hydroxybutyrate, and lactate in urinary organic acid analysis. Ultrasonography did not reveal any abnormalities. Suspecting metabolic disorder, the patient was treated with medium-chain triglyceride (MCT) milk and multiple vitamins (vitamins B1, B2, B12, C, E, biotin, CoenzymeQ10, and L-carnitine). This treatment was continued because his lactic acidosis improved.

**Figure 1:**
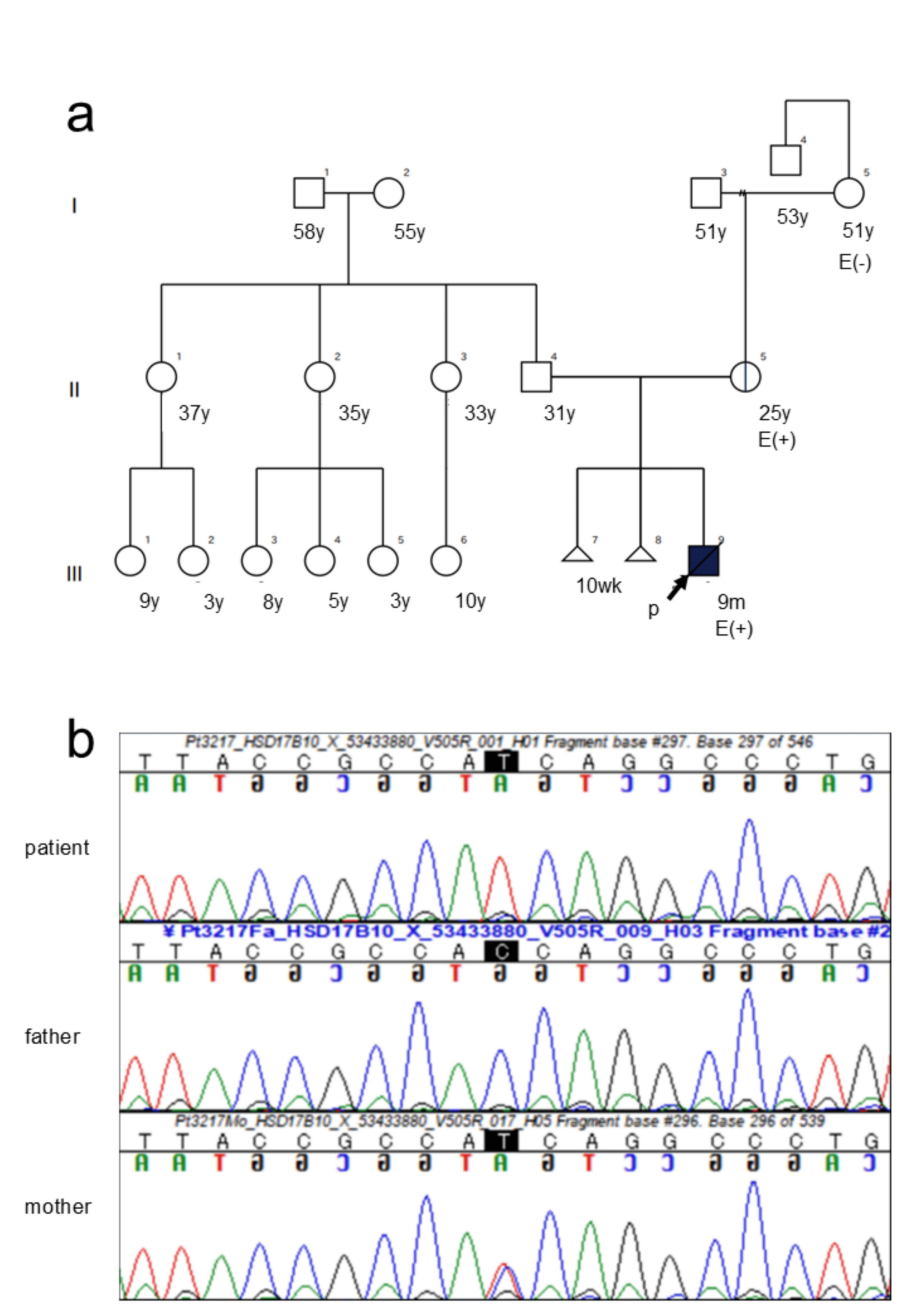
Pedigree and Sanger sequencing results. (a) The black arrow indicates the proband. Genetic testing was performed on the parents and the patient. The pedigree chart was created using novel, automated questionnaire-based software for pedigree chart creation [10]. (b) Sanger sequencing results for the patient, father, and mother are shown from top to bottom. The patient was hemizygous for the HSD17B10 c.34G>A p.(Val12Met) variant. His mother was a heterozygous carrier of the same variant, showing both the normal allele (G) and the mutated allele (A), while his father carried only the normal allele (G).

Genetic testing for metabolic diseases was performed using a targeted panel of nuclear and mitochondrial genes [8]. A hemizygous variant, NM_004493.3: c.34G>A p.(Val12Met) in HSD17B10, was identified, leading to the diagnosis of HSD10 disease. Based on the American College of Medical Genetics and Genomics (ACMG) and Association for Molecular Pathology (AMP) guidelines for sequence variant interpretation [9], we classified this variant as "likely pathogenic" because it met the criteria for PM1, PM2, PM5, PP2, PP3, and PP4. The patient's clinical phenotype was highly consistent with HSD10 disease. Furthermore, genetic testing of the patient's parents revealed that the mother, who had a mild intellectual disability and muscle weakness, was a heterozygous carrier of the same variant (Figures 1a, 1b).

**Figure 2:**
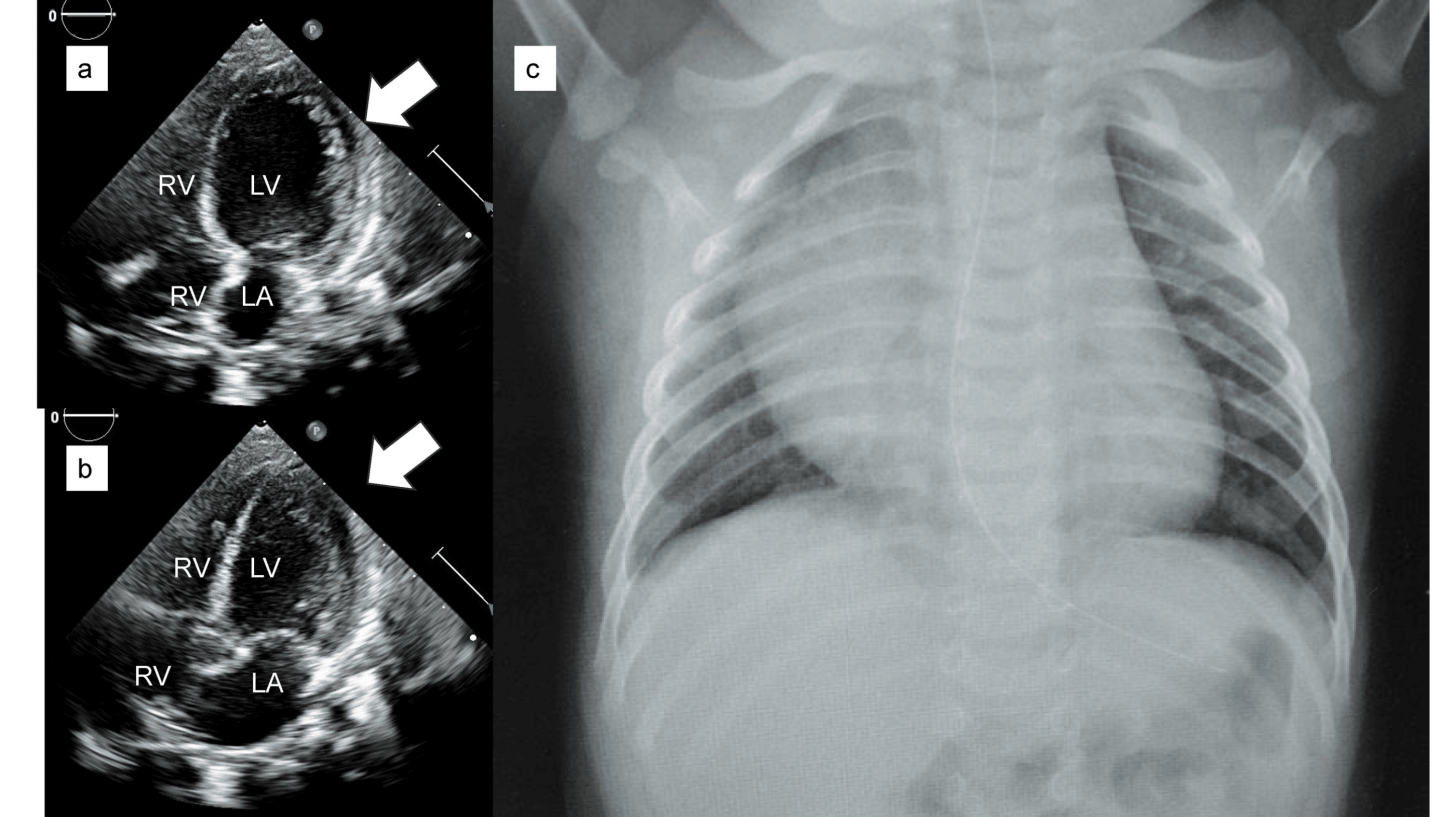
Transthoracic echocardiography and chest radiograph at five months. Echocardiography reveals no obvious left ventricular (LV, white arrowhead) enlargement, myocardial hypertrophy, or wall-motion abnormalities in four-chamber views during (a) diastole and (b) systole. (c) The chest radiograph shows no cardiac enlargement, with a cardiothoracic ratio of 55%. RV, right ventricle; RA, right atrium; LA, left atrium

At five months, he was referred to our center for evaluation of cardiac function. Echocardiography showed a visually estimated left ventricular ejection fraction (LVEF) of 60%, a left ventricular end-diastolic diameter (LVDd) of 28.0 mm (114% of normal), and a 55% cardiothoracic ratio (CTR) of the chest radiograph with no obvious left ventricular enlargement or myocardial hypertrophy (Figures 2a-c).

No delays in his physical development were detected, and his cardiac function and general condition remained stable. The patient received *Haemophilus influenzae* type b (Hib), diphtheria, tetanus, acellular pertussis, inactivated poliovirus (DTaP-IPV), and 20-valent pneumococcal conjugate vaccines at the age of six months. The BCG vaccine was administered nine days later. During an outpatient visit, 17 days after the initial vaccination, the patient presented with muscle weakness. Previously, he had been able to roll over, but could no longer do so, suggesting a regressive phenomenon.

**Figure 3:**
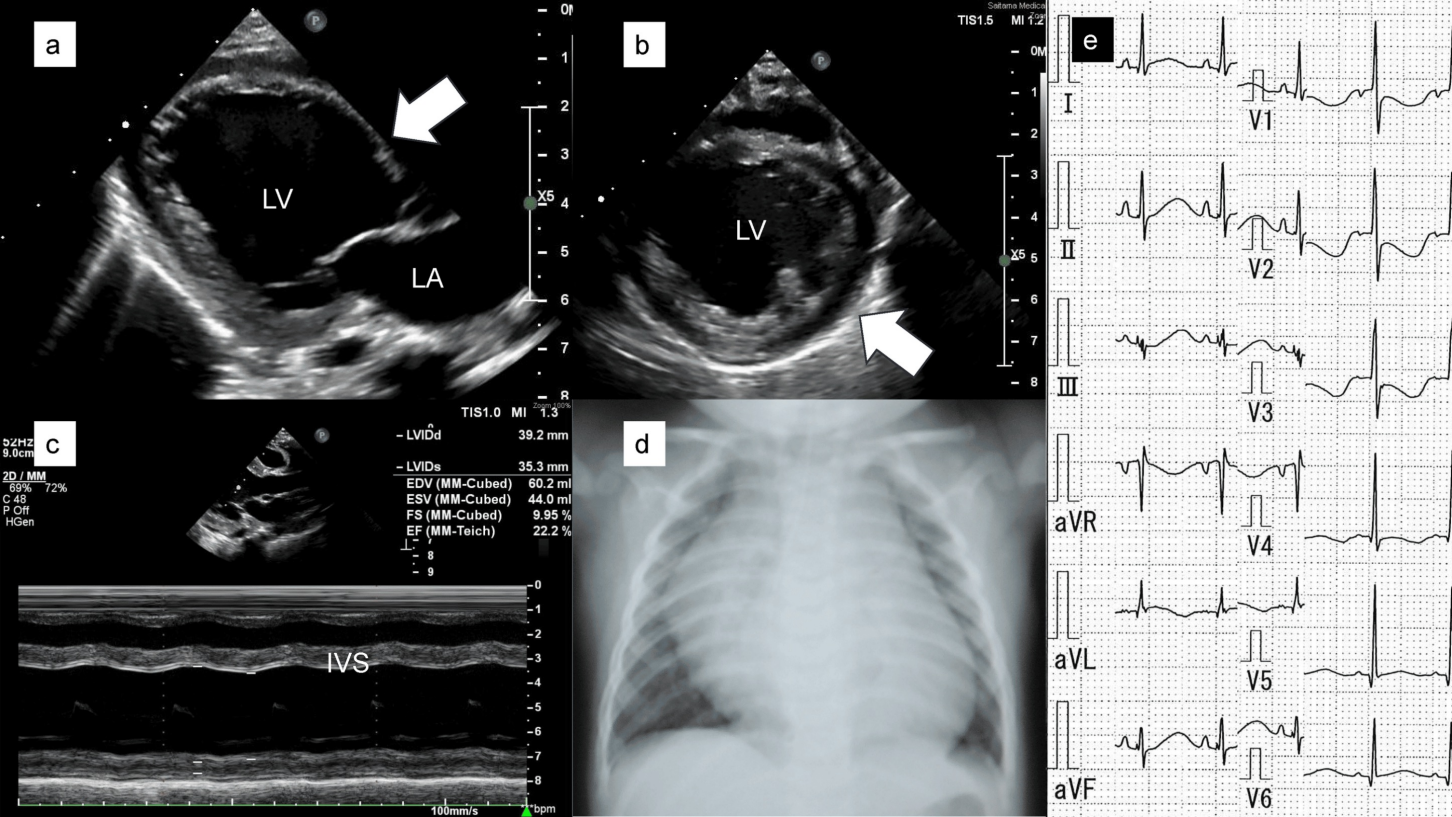
Transthoracic echocardiography, chest radiograph, and ECG at seven months. (a–c) Echocardiography of the left ventricle (LV, white arrowhead) (a) long- and (b) short-axis views, and (c) LV M-mode showing LV enlargement with reduced ejection fraction. (d) Chest radiograph shows cardiac enlargement with a cardiothoracic ratio of 65%. (e) Electrocardiogram reveals ST depression in leads between V1 and V3. LA, left atrium; IVS, interventricular septum

At seven months of age, the patient was admitted to our center with chief complaints of vomiting and nasal alar breathing. Echocardiography on admission showed an LVEF of 40%, which decreased to 22% 17 hours later, with left ventricular enlargement, LVDd of 39.2 mm (162% of normal), and no cardiac hypertrophy (Figures 3a-c). Chest radiography showed an increased CTR of 65% (Figure 3d), indicating cardiac enlargement. Electrocardiography (ECG) showed ST depression in leads V1 to V3 (Figure 3e).

Blood examination revealed elevated levels of creatine kinase (CK), CK-MB, troponin I, and BNP (Table 2).

**Table 2:** Initial laboratory testing on admission at seven months of age Blood tests revealed elevated creatine kinase (CK), CK-MB, troponin I, and BNP. Reference ranges were provided in the clinical context.

Parameter	Value	Reference Range	Unit
Creatine kinase (CK)	441	40–300	IU/L
CK-MB	35.8	0.0–5.0	ng/mL
Troponin I	536.6	0.0–26.2	pg/mL
BNP	4,190	0.0–18.4	pg/mL

The multiplex PCR panel for respiratory pathogens was positive for rhinovirus/enterovirus, leading to the diagnosis of viral myocarditis.

**Figure 4:**
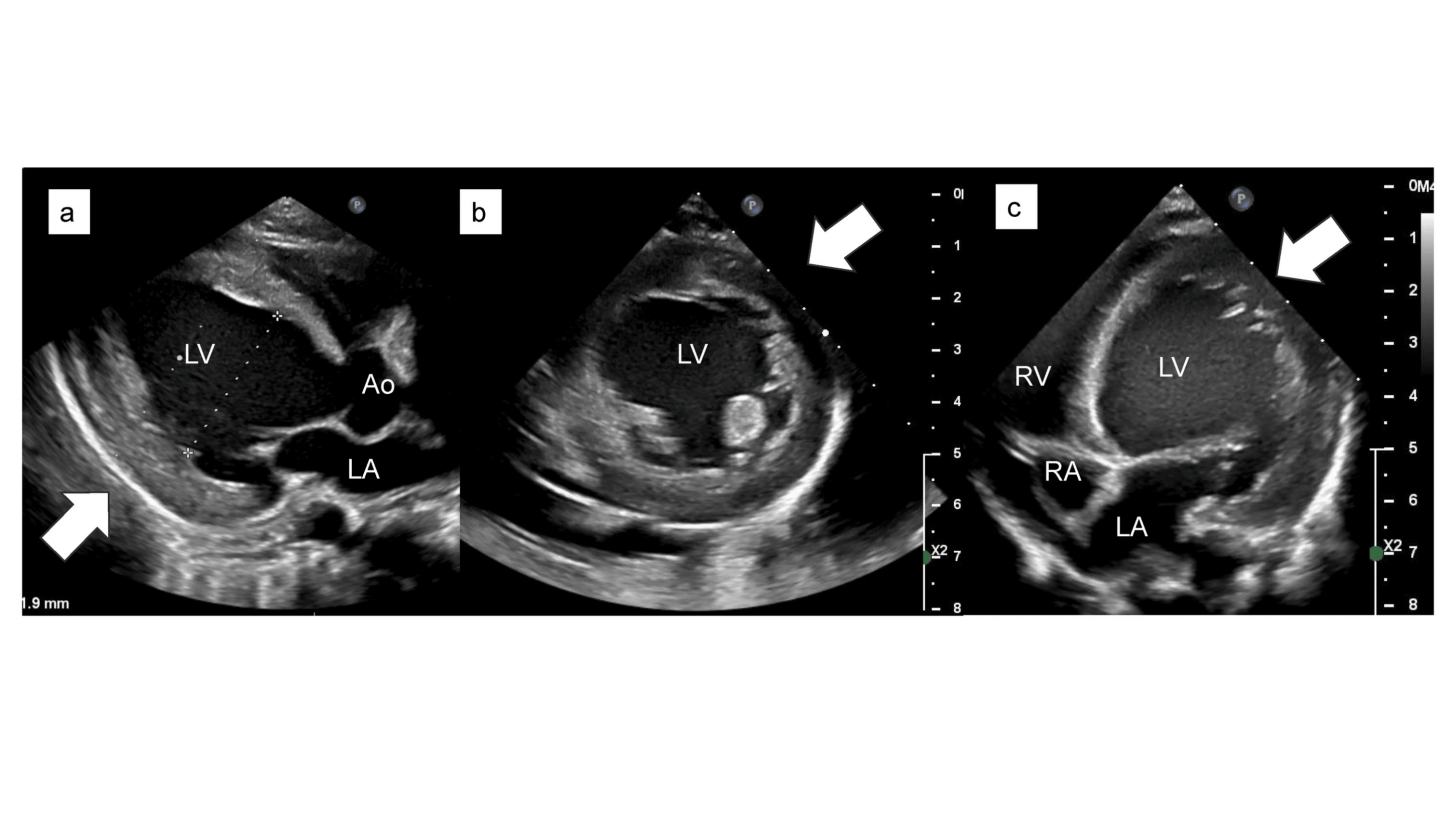
Transthoracic echocardiography at day four after hospitalization. (a–c) Echocardiography in the left ventricular (LV, white arrowhead) (a) long-axis, (b) short-axis, and (c) apical four-chamber views reveal LV enlargement with further reduced ejection fraction (EF) compared to those observed at admission. New-onset LV hypertrophy is observed. RV, right ventricle; RA, right atrium; LA, left atrium; Ao, aorta

After admission, LVEF on echocardiography did not improve and remained at approximately 20%. Subsequently, troponin I levels increased to 4,282 pg/mL and then became negative. Left ventricular enlargement persisted, and myocardial thickening was evident (Figures 4a-c).

ECG showed improvement of ST depression in V1 to V3 over time. Based on these findings, we diagnosed the patient with mixed hypertrophic and dilated cardiomyopathies.

Angiotensin-converting enzyme inhibitors and β-blockers were not initiated due to the nature of the underlying disease and concerns about potential side effects. He was discharged at eight months, but his cardiac function did not recover, and he developed pulmonary congestion and died at nine months.

## Discussion

The patient presented with hyperlactatemia and metabolic acidosis immediately after birth, and genetic testing for inborn errors in metabolism was performed. HSD10 with a previously unreported gene variant was rapidly diagnosed using panel testing and next-generation sequencing. The patient showed progressive neurodegeneration beginning after vaccination at about six months. He developed myocarditis and cardiomyopathy at seven months due to a viral infection and died at nine months.

Novel genetic variant

Approximately 40 case reports of HSD10 disease have been reported worldwide, and the clinical picture of this disease is still not fully understood [11]. Few reports are available on cases of the neonatal form of the disease. Only six cases of the neonatal form [7] of the Zschocke classification [1] have been reported (Table 3).

**Table 3:** Summary of genotypes and phenotypes in the neonatal form of HSD10 disease NA, not available

Patient	Variant	Sex	Onset	Clinical Feature	Family History	Present Status (Age)
1 (this report)	c.34G>A p.(Val12Met)	M	1 d	Metabolic acidosis, hyperlactatemia, hyperammonemia, hypoglycemia, hyperpnea	Mother has mental retardation	Dead (9 m)
2 [7]	c.59C>T p.(S20L)	M	2 d	Metabolic acidosis, development retardation, hyperlactatemia, hypoglycemia, Cholestatic hepatitis, elevated myocardial enzyme	Normal parents	Dead (3 m)
3 [12]	c.740A>G p.N247S	M	1 d	Metabolic acidosis, hypoglycemia, hypotonia, cyanosis, cardiomegaly, hyperlactatemia, and hyperlactaturia	Sister is patient, a normal mother	Dead (2 m)
4 [13]	c.740A>G p.N247S	M	1 d	Metabolic acidosis, mildly encephalopathic, hyperlactatemia, hyperlactaturia, hyperammonemia, feeding difficulties, PDA (patent ductus arteriosus), anemia, thrombocytopenia, elevated transaminases, coagulation	NA	Dead (6 m)
5 [14]	c.677G>A p.(R226Q)	M	1 d	Metabolic acidosis, developmental regression, hypoglycemia, anemia, hyperammonemia, thrombopenia, coagulation, hepatic dysfunction, myoclonus, seizures, hypertrophic cardiomyopathy	Normal mother	Dead (7 m)
6 [15]	c.677G>A p.(R226Q)	M	1 d	Metabolic acidosis, polypnea, moan, hypoglycemia, hyperlactatemia, psychomotor retardation	NA	NA
7 [6]	c.257A>G p.D86G	M	1 m	Neurodegeneration, progressive hypertrophic cardiomyopathy	NA	Dead (7 m)

In the present case, the identified genetic variant was novel and did not fit any of the previously reported categories. A total of 17 genetic variants have been reported as the cause of HSD10 disease [7]. The variants currently reported in neonatal patients include c.59C>T p.(S20L), c.740A>G p.N247S, c.677G>A p.(R226Q), and c.257A>G p. D86G. The variant in our case, NM_004493.3: c.34G>A p.(Val12Met), has a low allele frequency and is an unreported variant, whereas c.34G>C p.Val12Leu at the same position has been reported to be pathogenic based on functional verification [16]. The patient with the latter genetic variant, p. Val12Leu, showed developmental delay and hypotonia in infancy, and at 15 months, she developed a systolic heart murmur and was diagnosed with dilated cardiomyopathy. Subsequently, the patient developed poor cardiac function and died at 21 months [16]. Thus, the clinical course of patients with missense variants affecting the same amino acid residue is similar to that observed in our case.

Mechanisms of neurodegeneration and cardiomyopathy

The mechanism of neurodegeneration may involve multiple factors, as reported in previous studies.

First, neurodegeneration results from inhibition of isoleucine metabolism. Urinary organic acid analysis is one of the most important tests for the biochemical diagnosis of HSD10 disease. Elevated levels of 2M3HB and tiglylglycine were characteristic findings in this analysis [1]. These substances are intermediate metabolites of the isoleucine pathway, and 17β-HSD10 protein is a key enzyme in this pathway. When 17β-HSD10 is deficient, isoleucine metabolism is inhibited, and abnormal metabolites such as 2M3HB accumulation are excreted in the urine [17,18]. The accumulation of abnormal metabolites is neurotoxic and affects the nerve cells [19]. However, in the present case, urinary organic acid analysis showed elevated 2,3DH2MB, and the clinical findings of HSD10 disease may not be related to the accumulation of toxic metabolites in the isoleucine pathway [1].

Second, abnormalities in the metabolism of neuroactive steroids were observed. 17β-HSD10 is involved in the metabolism of allopregnanolone (ALLOP), an important neuroactive steroid in the brain. ALLOP is a positive allosteric modulator of gamma-aminobutyric acid type A (GABAa) receptors and is essential for normal GABAergic activity. Therefore, deficiency of 17β-HSD10 causes an imbalance in the metabolism of neurosteroids and GABAa modulators, leading to impaired brain development and neurological function [19].

The third mechanism involves the impairment of mitochondrial energy production. 17β-HSD10 acts as a component of mitochondrial ribonuclease P (RNase-P), which is important in the early steps of mitochondrial deoxyribonucleic acid (mtDNA) transcription and is involved in tRNA structure stabilization [20]. Mutations in the *HSD17B10* gene disrupt mtDNA transcription and inhibit tRNA maturation. Impaired tRNA processing disrupts mitochondrial RNase-P function, thereby reducing the activity and assembly of respiratory chain complexes I, III, IV, and V. This leads to reduced adenosine triphosphate (ATP) production, which limits the energy supply to neurons. Particularly in brain neurons, this energy deficit causes cognitive dysfunction and developmental regression, resulting in progressive neurodegeneration [13].

Similarly, abnormal metabolites such as 2M3HB that accumulate due to 17β-HSD10 deficiency may be toxic to cardiomyocytes [17]. The heart muscle requires large amounts of ATP for continuous contraction, and a lack of ATP production results in energy deficiency. This, in turn, causes cardiomyocyte death, prompting compensatory hypertrophy of the heart muscle, which eventually results in cardiomyopathy [13]. Cardiomyopathy is caused by a combination of factors, including decreased ATP production due to mitochondrial dysfunction, oxidative stress resulting from the excessive production of reactive oxygen species (ROS), and disruption of metabolic pathways. These abnormalities trigger remodeling reactions that ultimately lead to cardiomyopathy at the tissue level [21].

The causal relationship between mitochondrial dysfunction and the induction of myocarditis and cardiomyopathy remains unclear. Viral infection induces mitochondrial apoptosis in the myocardium and promotes the translocation of calpain-1, a cytosolic calcium-activated cysteine protease, into the mitochondria. This cascade of events leads to a further reduction in mitochondrial synthesis and an increase in ROS production [22]. These findings suggest that in patients with mitochondrial dysfunction due to 17β-HSD10 deficiency, viral infection may exacerbate the condition and lead to severe myocarditis and cardiomyopathy.

Vaccination was originally intended to prevent serious infectious diseases in children. However, the dilemma with this disease is that although the various symptoms of HSD10 can easily become severe due to viral infection, vaccination may trigger various symptoms such as neurodegeneration [1]. It is challenging to establish an effective treatment for HSD10 due to the limited number of patients with this disease and the numerous factors involved.

## Conclusions

This report describes a severe case of neonatal-onset HSD10 disease, caused by a novel genetic variant in the *HSD17B10* gene. A male infant presented with early metabolic issues, followed by neurodevelopmental regression at six months after vaccination. Rapidly progressive cardiomyopathy triggered by rhinovirus/enterovirus infection led to death at nine months. This aggressive deterioration highlights the poor prognosis of neonatal HSD10 disease, especially when triggered by viral infection.

This case, with its novel genetic variant and rapid, fatal cardiomyopathy, emphasizes the importance of clarifying genotype-phenotype correlations. However, this is a single case report, and the identified variant has not been functionally validated, which is required for definitive confirmation of its pathogenicity. While the observed neurodevelopmental regression followed vaccination, a direct causal relationship could not be established, as a coincidental occurrence could not be ruled out. To our knowledge, no prior studies have biochemically or physiologically elucidated a direct causal link between vaccination and neurodegeneration in HSD10 disease. Further accumulation of genotypes and clinical phenotypes is important to establish future treatment strategies.
